# Partitioning genetic and species diversity refines our understanding of species–genetic diversity relationships

**DOI:** 10.1002/ece3.4530

**Published:** 2018-12-11

**Authors:** Vera Wilder Pfeiffer, Brett Michael Ford, Johann Housset, Audrey McCombs, José Luis Blanco‐Pastor, Nicolas Gouin, Stéphanie Manel, Angéline Bertin

**Affiliations:** ^1^ Nelson Institute for Environmental Science University of Wisconsin – Madison Madison Wisconsin; ^2^ Department of Biology University of British Columbia Kelowna British Columbia Canada; ^3^ Alcina Forets Montpellier France; ^4^ Centre d’étude de la forêt Université du Québec à Montréal Montréal Quebec Canada; ^5^ Department of Statistics, Ecology and Evolutionary Biology Program Iowa State University Ames Iowa; ^6^ INRA Centre Nouvelle‐Aquitaine‐Poitiers UR4 (URP3F) Lusignan France; ^7^ Departamento de Biología Facultad de Ciencias Universidad de La Serena La Serena Chile; ^8^ Centro de Estudios Avanzados en Zonas Áridas La Serena Chile; ^9^ Instituto de Investigación Multidisciplinar en Ciencia y Tecnología Universidad de La Serena La Serena Chile; ^10^ EPHE PSL Research University CNRS UM, SupAgro, IRD INRA UMR 5175 CEFE Montpellier France

**Keywords:** genetic outlier, high Andean wetlands, SNP, species–genetic diversity correlation

## Abstract

Disentangling the origin of species–genetic diversity correlations (SGDCs) is a challenging task that provides insight into the way that neutral and adaptive processes influence diversity at multiple levels. Genetic and species diversity are comprised by components that respond differently to the same ecological processes. Thus, it can be useful to partition species and genetic diversity into their different components to infer the mechanisms behind SGDCs. In this study, we applied such an approach using a high‐elevation Andean wetland system, where previous evidence identified neutral processes as major determinants of the strong and positive covariation between plant species richness and AFLP genetic diversity of the common sedge *Carex gayana*. To tease apart putative neutral and non‐neutral genetic variation of *C. gayana*, we identified loci putatively under selection from a dataset of 1,709 SNPs produced using restriction site‐associated DNA sequencing (RAD‐seq). Significant and positive relationships between local estimates of genetic and species diversities (α‐SGDCs) were only found with the putatively neutral loci datasets and with species richness, confirming that neutral processes were primarily driving the correlations and that the involved processes differentially influenced local species diversity components (i.e., richness and evenness). In contrast, SGDCs based on genetic and community dissimilarities (β‐SGDCs) were only significant with the putative non‐neutral datasets. This suggests that selective processes influencing *C. gayana* genetic diversity were involved in the detected correlations. Together, our results demonstrate that analyzing distinct components of genetic and species diversity simultaneously is useful to determine the mechanisms behind species–genetic diversity relationships.

## 1 INTRODUCTION

The mechanisms that produce and maintain diversity spark both theoretical and practical interest across ecology and evolutionary biology. Yet, historical separation between genetic and organismal ecology research has limited the development of a cohesive framework for multilevel analysis (Antonovics, 2003). Recently, however, researchers have integrated these domains, investigating possible correlations between the genetic diversity of a focal species and the species diversity of the associated community, deepening the description of the distribution of biodiversity, and improving our understanding of community assembly (Antonovics, 2003; Lamy, Laroche, David, Massol, & Jarne, 2017; Laroche, Jarne, Lamy, David, & Massol, 2015; Vellend & Geber, 2005; Vellend et al., 2014; Whitham et al., 2006; Whitlock, 2014). In theory, various evolutionary mechanisms can drive positive or negative covariation between genetic and species diversity, including both neutral (e.g., drift, immigration) and adaptive (e.g., selection) processes (Lamy et al., 2017; Vellend & Geber, 2005). Thus, investigating the parallels between species and genetic diversity may help synthesize concepts from multiple divisions of biodiversity research and connect different perspectives in ecology and evolution.

Neutral processes can have analogous effects on both genetic and species diversity (Chave, 2004; Etienne & Olff, 2004; Vellend & Geber, 2005; but see Laroche et al., 2015) and consequently can create positive species–genetic diversity correlations (SGDCs). Island biogeography theory predicts that species richness will go up as habitat area and connectivity increase (MacArthur & Wilson, 1967; Rosenzweig, 1995), and population genetic theory predicts identical genetic responses in allele diversity to these same structural elements of habitat (i.e., habitat area and connectivity) (Kimura, 1983; Wright, 1931). Recent evidence suggests that neutral processes play a dominant role in positive species–genetic diversity relationships (Lamy et al., 2013; Odat, Jetschke, & Hellwig, 2004; Papadopoulou et al., 2011; Struebig et al., 2011; Vellend, 2004; Vellend et al., 2014). Positive SGDCs are most common where neutral processes including migration, drift, and demographic stochasticity are expected to have a particularly strong influence on both diversity levels. For instance, a recent review (Vellend et al., 2014) of 40 empirical studies that estimated 115 SGDCs found that systems with discrete, isolated habitat patches almost always show a positive correlation between species diversity and genetic diversity (see also Laroche et al., 2015; and Whitlock, 2014), contrary to what is observed in nonfragmented habitats.

SGDC studies have traditionally focused on neutral genetic diversity, although adaptive diversity has been occasionally considered (Bertin et al., 2017; Kahilainen, Puurtinen, & Kotiaho, 2014; Vellend et al., 2014; Watanabe & Monaghan, 2017). While neutral processes affect the whole genome uniformly, selection acts on specific regions, which bear the footprint of selection (Holderegger, Kamm, & Gugerli, 2006). Thus, as long as the effects of selection are not completely overridden by neutral processes influencing the whole genome (i.e., high gene flow or drift levels, for instance), adaptive genetic diversity will show deviating patterns from neutral genetic diversity. With the advent of next‐generation sequencing, it is now possible to distinguish patterns generated by neutral evolutionary forces and adaptive processes (Balkenhol, Cushman, Storfer, & Waits, 2015; Batista, Janes, Boone, Murray, & Sperling, 2016; Meyer‐Lucht et al., 2016) by investigating both neutral and adaptive genetic diversity separately. In SGDC studies, this approach can provide a clarified portrayal of similarity in the role of neutral processes on species and genetic diversity, and can thus help determine whether neutral processes are participating in the production of species–genetic diversity correlations. Two recent studies have applied this approach (Bertin et al., 2017; Watanabe & Monaghan, 2017). Bertin et al. (2017) demonstrated that AFLP loci putatively under selection (i.e., outlier loci) decreased overall genetic diversity and decreased the strength of the correlation between plant richness and genetic diversity across five high Andean wetland species, suggesting that the neutral and adaptive components of genetic diversity covary differently with species diversity. Similarly, Watanabe and Monaghan (2017) found deviating relationships between stream macroinvertebrate species and genetic diversity for putatively neutral loci versus loci under selection. However, because both Bertin et al. (2017) and Watanabe and Monaghan (2017) used AFLP markers, only a few loci putatively under selection were identified (an average of eight across all species’ datasets). Because genetic diversity estimates are sensitive to the number of genetic markers (Dutoit, Burri, Nater, Mugal, & Ellegren, 2017), such a low number of outlier loci is insufficient to calculate robust genetic diversity estimates of non‐neutral diversity. Furthermore, to effectively compare neutral and non‐neutral loci patterns, an equal number of both types of loci should ideally be used (Batista et al., 2016).

Here, we deepen and expand Bertin et al. (2017) and Watanabe and Monaghan (2017)'s comparative approaches by partitioning both genetic and species diversity and considering both site‐level (α‐diversity) and landscape‐level (β‐diversity) diversity. Contrary to genetic diversity, species diversity cannot be separated into its neutral and non‐neutral attributes, but it is comprised of various dimensions that respond differently to the same ecological processes (Biswas, MacDonald, & Chen, 2017; Stirling & Wilsey, 2001). For instance, evidence indicates that dispersal and competition differentially affect the local diversity indices (α‐diversity), with dispersal being of greater relevance for species richness and competition for species evenness (Stirling & Wilsey, 2001). Incorporating the different facets of α‐diversity in SGDC studies can thus broaden the insights achieved regarding the ecological processes that contribute to correlations between species and genetic diversity. Similarly, several authors recently called for extending correlation analysis between local genetic and species diversities (α‐SGDC, Kahilainen et al., 2014) to landscape scales by investigating the correlation between genetic and species dissimilarities (β‐SGDC, Kahilainen et al., 2014) as a means to improve our understanding of community assembly (Lamy et al., 2017) and biodiversity variation at landscape scales (Kahilainen et al., 2014).

In this study, we focused on the species–genetic diversity relationship between a high Andean plant community and the herbaceous grass‐like plant *Carex gayana* in Chile's Norte Chico. This system is ideal for the proposed framework since: (a) Previous evidence suggests that neutral processes, dispersal in particular (e.g., isolation by distance), cause genetic structure in *C. gayana* (Troncoso, Bertin, Osorio, Arancio, & Gouin, 2017) and a strong and positive SGDC between plant richness and genetic diversity of *C. gayana* (*r *=* *0.60, *p *<* *0.05 according to Bertin et al., 2017). Bertin et al. (2017) found that the SGDC did not hold when the effects of wetland connectivity, which explained about 50% of the variation in both diversity components, were factored out (*r *=* *0.25, *p *>* *0.05). (b) High Andean wetlands in this region are highly fragmented and experience highly variable environmental conditions due to large‐scale climatic variations and local abiotic fluctuations resulting from the sharp orography of the Andes. As a result, we expect the footprint of selection on adaptive genetic variation of high Andean wetland populations to be strong and to cause significant deviating patterns between neutral and adaptive genetic diversity.

## 2 MATERIALS AND METHODS

### 2.1 Study system

*Carex gayana* is an herbaceous perennial sedge species of the Cyperaceae family inhabiting high‐elevation wetlands of the Andes Mountains. It is monoecious and displays both sexual reproduction and vegetative propagation through small rhizomes (Troncoso et al., 2017). Sedges frequently dominate extensive areas and play a particularly important role in wetlands. The ploidy level of *C. gayana* is unknown; however, polyploidy is rare in the *Carex* genus (Lipnerová, Bureš, Horová, & Šmarda, 2013). Genetic diversity levels found in our study and initial evidence from AFLP markers (Troncoso et al., 2017) are more similar to those found in diploid than in polyploid plant species (Hoeltgebaum & dos Reis, 2017; Kim, Shin, & Choi, 2009; Sampson & Byrne, 2012).

**Figure 1:**
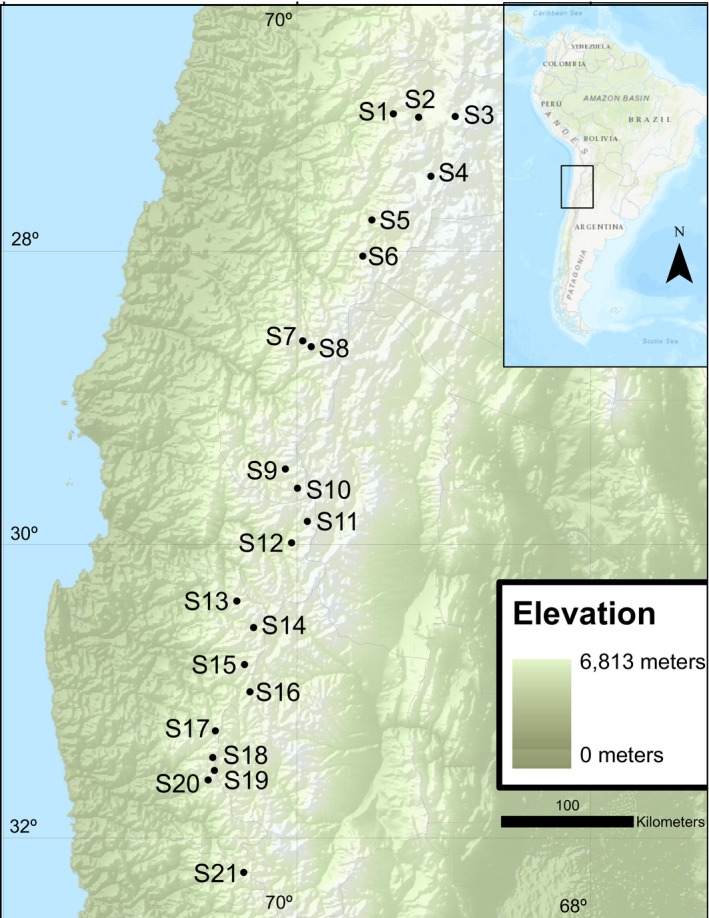
Location of the 21 high Andean wetlands sampled in Chile's Norte Chico by Bertin et al. (2017) and Troncoso et al. (2017). Sites 13 (no genetic information), 2, and 4 (excluded following SNP data filtering) were not included in this study

In the high Andes Mountains, sedge‐dominated wetlands are interspersed throughout an arid grassland matrix, spread along a latitudinal gradient characterized by high aridity at lower latitudes. High Andean wetlands are fed by glacial melt and ground upwelling with high wetland density at high elevations (Squeo, Warner, Aravena, & Espinoza, 2006). Study sites were located between 2,852 and 4,307 meter elevations, across a 600‐kilometer stretch of the Andes north of Santiago, Chile (Figure 1), over which a large climatic gradient occurs, with mean annual precipitation ranging between 35 and 200 mm for the northernmost and southernmost limits of the study zone. Based on a previous analysis of the genetic structure of the populations under study (Troncoso et al., 2017), we considered each site as a separate population.

### 2.2 Sampling, DNA extraction, and next‐generation sequencing

Species sampling and DNA extraction procedures have been previously described in Bertin et al. (2017) and Troncoso et al. (2017). We selected between 4 and 10 samples per site from 20 wetlands based on DNA quality and quantity (Table 1), for a total of 190 samples, which were sent to the Genomic Diversity Facility (GDF; http://www.biotech.cornell.edu/brc/genomic-diversity-facility) at Cornell University for genotyping‐by‐sequencing (GBS; Elshire et al., 2011). Samples from site 13 (Figure 1) used by Bertin et al. (2017) and Troncoso et al. (2017) were not included in this study because DNA concentrations did not meet GDF requirements. Three restriction enzymes were tested for GBS library construction (a four‐base cutter: *Ape*KI, and two‐six‐base cutters: *Eco*T22I and *Pst*I). *Pst*I was not retained because the amplified library presented adapter dimer peaks and a smaller fragment size distribution. Although the four‐base cutter *Ape*KI generated the library with the largest fragment pool, we selected the six‐base cutter *Eco*T22I because *C. gayana* genome size and genetic diversity level were not known, hence ensuring a higher coverage per SNP locus due to the lower proportion of amplified fragments. Libraries were multiplexed with 95 samples assigned to each of two lanes and sequenced on an Illumina HiSeq 2000 platform as single‐end, 100 base pair reads. Lane 1 was composed mainly of samples from the Copiapo, Choapa, and Elqui river basins, and lane 2 mainly of samples from the Elqui, Huasco, and Limarí basins (see map in Bertin et al. (2017) for basin corresponding with each population in Table 1). Although the number of detected tags differed between lanes 1 and 2 (18,129,903 and 13,413,636, respectively), the average proportion of missing data per sample in the postfiltered data (see section below) appeared relatively similar (3.8% and 6.0%, respectively), indicating no particular bias in our data.

**Table 1:** Expected heterozygosity (*He*) of *Carex gayana* populations calculated from the five SNP datasets (DS1–5, see Table 2) and from AFLP data, as well as plant species richness at each site. DS1 is the full, original dataset, DS2 and DS3, the two non‐outlier datasets, and DS4 and DS5 the two outlier datasets. Populations with conspicuously low SNP genetic diversity in comparison with their AFLP genetic diversity (see Supporting Information Figure S3) appear in bold

Population	Name	*n*	Species richness	Expected heterozygosity (*He*)					
				DS1	DS2	DS3	DS4	DS5	AFLP
S1	Cop4	4	11	0.072	0.071	0.073	0.080	0.043	0.070
S5	Cop5	10	14	0.084	0.082	0.084	0.097	0.088	0.068
**S6**	**Cop6**	**9**	**19**	**0.088**	**0.090**	**0.089**	**0.072**	**0.019**	**0.131**
S7	Hua3	10	16	0.134	0.143	0.136	0.080	0.041	0.095
S8	Hua2	9	17	0.127	0.136	0.129	0.069	0.052	0.075
S9	Hua1	10	16	0.122	0.129	0.124	0.076	0.018	0.082
S10	Elq3	10	15	0.114	0.120	0.117	0.077	0.015	0.091
S11	Elq4	10	10	0.159	0.163	0.161	0.136	0.082	0.113
S12	Elq2	10	11	0.130	0.124	0.129	0.165	0.175	0.089
S14	Lim3	9	19	0.145	0.152	0.148	0.097	0.018	0.105
S15	Lim4	10	19	0.143	0.152	0.146	0.081	0.028	0.097
S16	Lim1	9	15	0.099	0.108	0.101	0.042	0.014	0.077
S17	Lim2	9	19	0.134	0.144	0.136	0.068	0.046	0.111
S18	Cho3	10	21	0.197	0.205	0.201	0.143	0.042	0.170
S19	Cho2	9	20	0.219	0.227	0.224	0.169	0.018	0.254
S20	Cho1	10	17	0.175	0.180	0.177	0.140	0.055	0.187
**S21**	**Cho4**	**10**	**19**	**0.062**	**0.060**	**0.062**	**0.076**	**0.102**	**0.137**
	Mean (±*SD*)	9.3 (1.4)	16.4 (3.3)	0.130 (0.042)	0.135 (0.045)	0.132 (0.044)	0.098 (0.038)	0.050 (0.042)	0.115 (0.049)

### 2.3 Genetic data and bioinformatics

The UNEAK GBS pipeline (Lu et al., 2013), a method specially tailored for species that lack a reference genome, was used for SNP discovery. The UNEAK pipeline is a component of the TASSEL 3.0 bioinformatics package (Bradbury et al., 2007) that calls SNPs after resolving artifacts of problematic data including repeats, paralogs, and sequencing errors. These analyses were carried out by the GDF staff at Cornell University and are summarized in Supporting Information Table S1. Briefly, sequencing errors were filtered out by retaining only tags that were present at least 3 times, using a maximum error tolerance rate of 0.03 in the network filter (filters on the identification of reciprocal tag pairs), accounting for 0.01 average sequencing error rate to decide between homozygous and heterozygous calls, and setting minimum minor allele frequency (MAF) to 0.01. To avoid paralogs, a value of 0.05 was used as threshold mismatch rate above which the duplicate SNPs were not merged. This is a conservative threshold given the recommendations of using a value of 0.1 for species with high residual heterozygosity. We then used VCFtools (Danecek et al., 2011) to further filter the SNPs output from the UNEAK pipeline (See Supporting Information Table S2 for detailed outline of remaining sites after each filtering step). We first applied a minimum depth filter of 10 reads to exclude all genotypes with insufficient coverage by treating them as missing data. We then used a histogram of mean site depth to determine the appropriate parameters for maximum mean site depth, excluding sites with high (>50 reads) representation based on the shape of the distribution. After filtering for site depth, we retained all loci with less than 40% missing data. The amount of missing data retained for all SNPs may have serious consequences on values of genetic diversity and calculations of SGDCs; therefore, we reran all analyses with a more stringent dataset, retaining all loci with less than 30% missing data. The more stringent dataset provided very similar results (not shown), and therefore, we chose to retain the 40% missing data filter to provide a greater representation of the genome. To remove potential sequencing errors, we chose a MAF of 0.04 for all loci across all individuals. Furthermore, we only included loci that were biallelic. We calculated the observed heterozygosity for all remaining sites and excluded sites with observed heterozygosity >0.5 to exclude potential paralogs (Hohenlohe, Amish, Catchen, Allendorf, & Luikart, 2011). Call rate, the number of sites successfully genotyped for each individual, was calculated per individual, and any individual with lower than 40% call rate was removed from the dataset prior to further analysis. To check for the presence of clonal individuals, we calculated the observed number of multilocus genotypes using the mgl function of the “poppr” in R (Kamvar et al., 2018). No redundant multilocus genotypes were identified, which indicates that no clones were present in our dataset. All loci were then tested for departure from Hardy–Weinberg equilibrium using the hw.test function of the “pegas” package in R (Paradis et al., 2013). Significance was tested using 1,000 random permutations.

### 2.4 Environmental data

Ten environmental variables were used to test for genome–environment–associations: mean annual precipitation (MAP), mean average wind speed (MAWS), number of days with snow cover (SnowNDays), mean annual temperature (MAT), soil moisture (TCI), slope, aspect, productivity (NDVI), and two independent estimates of wetland surface. MAP was estimated from average monthly precipitation (mm), calculated by interpolation of precipitation gauge network measurements over the 32‐year period from 1975 to 2006 (see details in Bertin et al., 2015). MAWS, in meters per second, was estimated over a 16‐year period using the nonhydrostatic Karlsruhe Atmospheric Mesoscale Model (see details in Bertin et al., 2015). SnowNDays was calculated over a 12‐year period (2000–2011) from daily estimates of snow cover obtained from 500 m resolution MODIS satellite imagery in Google Earth Engine Explorer. MAT was assessed for each wetland using the high‐resolution gridded database of WorldClim (Hijmans, Cameron, Parra, Jones, & Jarvis, 2005). Topographic Convergence Index (TCI) was calculated in ArcGIS 10.0 using an ASTER Global Digital Elevation Model (GDEM) to calculate slope and upslope accumulating area. Slope was measured from the ASTER Global Digital Elevation Model (DEM) in Google Earth Engine Explorer. Aspect was recorded as a categorical variable with two categories: S/SE aspects or N/W/SW aspects. Normalized Difference Vegetation Index (NDVI) was calculated based on 30 m resolution LandSat 8 OLI satellite images from NASA obtained from the United States Geological Survey website (http://glovis.usgs.gov/). The wetland surface estimates were calculated in Google Earth Engine Explorer based on Google Earth surface1 and surface2 from NDVI.

To identify environmental predictors that were potentially collinear, we calculated Pearson correlations between each environmental variable pair and discarded variables that displayed repeated correlations exceeding 0.7. The final dataset included MAP, MAWS, MAT, TCI, slope, aspect, and NDVI.

### 2.5 Outlier detection

All the statistical and outlier detection analyses were performed in the R environment (https://cran.r-project.org). To identify loci deviating from neutral expectations, we combined two individual‐based approaches: one centered on the identification of outlier loci with respect to population structure and the second on genotype–environment associations. For the first approach, we used pcadapt (Duforet‐Frebourg, Bazin, & Blum, 2014; Luu, Bazin, & Blum, 2017), an outlier detection method that identifies loci putatively under positive local selection. Because such loci tend to increase genetic differentiation, pcadapt considers loci that contribute significantly more to population structure than most loci as candidate markers. To identify such loci, pcadapt uses a two‐step procedure. First, a principal component analysis captures the genetic structure of the dataset. Then, the Mahalanobis distance of the z‐scores on the first k‐components of each locus detects those loci that most relate to population structure (Luu et al., 2017). Here, we identified the optimal number of components (i.e., k‐components) from the scree plot and used a 10% false discovery rate to identify outlier loci with significantly larger Mahalanobis distances.

For our second approach, we identified SNP loci associated with environmental variables using redundancy analysis (RDA), a genome–environment association (GEA) approach to distinguishing candidate loci under selection based on correlation between genotype and environmental factors expected to impose natural selection. RDA is a canonical ordination technique where, first, response variables (multiple loci) are modeled as a function of linear combinations of the predictors (multiple environmental variables), then a PCA of the fitted values produces the RDA components that best explain, in sequential order, the variation among the fitted genetic values (Forester, Jones, Joost, Landguth, & Lasky, 2016; Legendre & Legendre, 2012; Talbot et al., 2017). To check that the final model did not suffer multi‐collinearity problems, we calculated the variance inflation factors (VIFs) and verified that none of them exceeded 5 for any of the predictors. Outlier loci were defined as those that were strongly influenced by the environmental variables, according to their *z*‐scores on the first RDA components (i.e., z‐scores exceeding twice the interquartile range; Forester et al., 2016). The number of components considered in this procedure was determined by examining the inertia scree plot and by verifying that all the selected components were significant. The RDA was performed following the methods described in Borcard, Gillet, and Legendre (2011) using the R package vegan (Oksanen et al., 2014). Significance of each individual RDA axis was tested with ANOVA‐like permutation tests with 9,999 randomizations. We calculated correlations between the outliers for each significant axis and the environmental variables to identify which variables may be having the greatest impact on non‐neutral patterns in our study system.

### 2.6 The genetic datasets

To test for correlations between species and genetic diversity, we created five SNP datasets. The expected composition for these datasets is reported in Table 2. DS1, the original dataset after filtering, included all SNPs and thus a large proportion of neutral loci and some non‐neutral ones. The nonoutlier datasets, DS2 and DS3, were formed by excluding either all the outlier loci identified (DS2), or only those jointly identified by the two outlier detection methods (DS3). The two datasets are thus composed for the most part of neutral loci, with a greater number of false negatives (adaptive loci) being expected in DS3 than in DS2. DS4 and DS5 contained the loci putatively under selection identified by at least one outlier detection method and by both detection methods, respectively. DS5 thus only included SNP loci for which we had convergent evidence of deviations from neutral expectations. In DS4 and DS5, the proportion of non‐neutral loci was expected to be much higher than in any other datasets. Some false positives (neutral loci) are likely to be present as well, but much less so in DS5 than in DS4.

**Table 2:** Expected and observed α and β SGDCs between species diversity and genetic diversity of the SNP loci for different *Carex gayana* genetic datasets (DS1–DS5). Gray parts of the Venn diagrams indicate SNP ensembles included in the dataset

	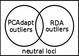 Datasets				
	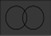 DS1	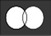 DS2	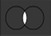 DS3	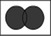 DS4	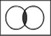 DS5
Dataset composition	Mainly neutral loci and some non‐neutral loci	Mainly neutral loci and fewer non‐neutral loci (false negatives) than DS1 and DS3	Mainly neutral loci and fewer non‐neutral loci (false negatives) than DS1	Adaptive loci and some false positives (neutral loci)	Adaptive loci and less false positives (neutral loci) than DS4
Expected signal of neutral processes	Strong	Strong, stronger than in DS1 and DS3	Strong, stronger than in DS1	Weak to moderate, much weaker than in DS1, DS2 and DS3	Weak to moderate, much weaker than in DS1, DS2, and DS3, and weaker than in DS4
Expected SGDC if neutrally driven	r_DS1_ > 0	r_DS2_ > 0	r_DS3_ > 0	r_DS4_ ≥ 0	r_DS5_ ≥ 0
		r_DS2_ > r_DS1_	r_DS3_ > r_DS1_	r_DS4_ < r_DS2_	r_DS5_ < r_DS2_
			r_DS2_ > r_DS3_	r_DS4_ < r_DS3_	r_DS5_ < r_DS3_

Number of loci	1,709	1,480	1,670	229	39
1. α‐SGDC					
with species richness					
17 sites					
α‐SGDC	0.33	0.37	0.34	−0.08	−0.47
*p*	0.01	0.07	0.09	0.62	0.97
15 sites					
α‐SGDC	0.57	0.6	0.58	−0.09	−0.56
*p*	0.01	<0.01	0.01	0.51	0.97
with species evenness					
17 sites					
α‐SGDC	−0.07	−0.05	−0.07	−0.24	−0.17
*p*	0.79	0.86	0.80	0.34	0.50
15 sites					
α‐SGDC	−0.02	0.01	−0.02	−0.24	−0.25
*p*	0.93	0.98	0.95	0.39	0.37
2. β‐SGDC					
17 sites					
β‐SGDC	0.15	0.11	0.13	0.26	0.21
*p*	0.10	0.16	0.12	0.02	0.05
15 sites					
β‐SGDC	0.16	0.14	0.16	0.26	0.27
*p*	0.08	0.12	0.09	0.01	0.01

When species diversity and genetic diversity correlations are neutrally driven, the relative proportion of the neutral and non‐neutral loci in the genetic dataset will condition the strength of the SGDC. Based on this rationale, we anticipated neutrally driven SGDCs to be positive for the putatively neutral loci, and to be the strongest in DS2, as DS2 is the more conservatively filtered neutral dataset (Table 2). Because the fraction of neutral loci is lower in DS4 and DS5 than in the putatively neutral datasets, these two datasets should display weaker correlations, with either a null or weakly positive association depending on the number of false negatives (neutral loci) in the dataset and the footprint of the neutral processes on the non‐neutral loci. When species diversity and genetic diversity correlations are driven by non‐neutral (i.e., adaptive) processes, we expect the SGDCs either to be null for all the datasets or to be only positive in DS4 and/or DS5, depending if the adaptive loci involved in the species–genetic diversity relationship are included in these SNP datasets.

### 2.7 Genetic diversity and species diversity

Before performing genetic diversity estimations, we first investigated the effects of within‐population missing data. Genetic estimates of α‐diversity were calculated for each population after varying the minimum number of individuals genotyped at each locus from four to eight individuals. Overall, we did not observe an influence of the number of individuals on the genetic diversity estimates (Supporting Information Figure S1). Therefore, we decided to consider all loci with a minimum of four genotyped individuals per population, which allowed us to calculate a genetic diversity estimate for all sites, including site 1, which only had four genotyped individuals. We evaluated within‐population genetic diversity for all populations and datasets as the expected heterozygosity (*He*) over the loci using gstudio (Dyer, 2017) as a measure of α‐diversity for the genetic datasets. To check the importance of the neutral signal in the non‐neutral loci datasets (due either to the presence of false positives or a strong influence of neutral processes on the whole genome), we calculated the pairwise Pearson correlations between the *He* estimates of each dataset.

Genetic β‐diversity was measured as the Cavalli‐Sforza genetic distance, calculated as

$$D_{\mathrm{CH}}=\frac{2}{\pi }\sqrt{2(1-\sum_{l}\sum_{u}\sqrt{X_{u}Y_{u}}},$$

where *X* and *Y* represent two populations for which *L* loci have been studied. $X_{u}$ represents the *u* ^th^ allele at the *l* ^th^ locus (Cavalli‐Sforza & Edwards, 1967).

The plant species assemblage was surveyed in each wetland based on five 30 × 30 cm quadrats. Details about the sampling strategy and method can be found in Bertin et al. (2017). Briefly, the length of each wetland was divided into five sectors and a quadrat was randomly placed within each sector. Plant species were then separated and identified in the laboratory, and their biomass (g/m^2^) evaluated after complete drying of the vegetal material in an oven at 70°C. Species α‐diversity was evaluated for each wetland as species richness (*S*) accumulated over the five quadrats and as species evenness using Pielou's evenness metric,

$$J=\frac{H^{\prime }}{H_{\max}}$$

where *H*′ is the Shannon diversity of the community calculated from plant abundances measured as their dry biomass across the five quadrats, and $H_{\max}=\ln S$, the maximum value of the Shannon diversity if every species was equally represented (McCune, Grace, & Urban, 2002). To estimate species β‐diversity, we calculated Bray–Curtis dissimilarity of the plant communities based on $\log_{10}\left(x+1\right)$ of the plant abundances, based on the equation,

$${\text{BC}}_{\mathrm{ij}}=1-\frac{2C_{\mathrm{ij}}}{S_{i}+S_{j}}$$

where $C_{\mathrm{ij}}$ is the sum of the lesser abundance values of only the species that occur at both sites, while $S_{i}$ and $S_{j}$ are the total number of species counted at each site (Legendre & Legendre, 2012).

### 2.8 Species–genetic diversity correlations between the SNP datasets

**Figure 2:**
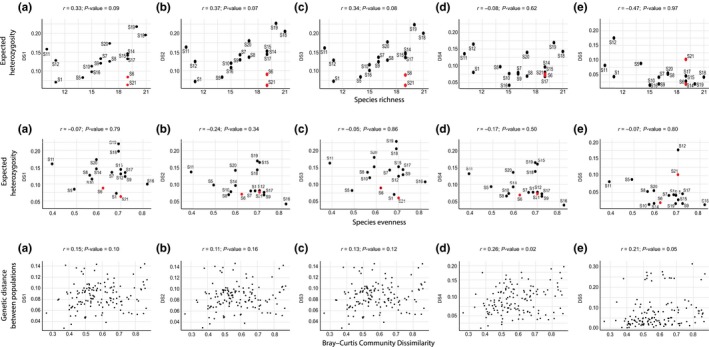
α‐Genetic diversity of *Carex gayana* plotted against site level species richness (top row) and evenness (middle row) and β‐genetic diversity plotted against β‐species diversity (bottom row) for the 17 sites included in this study using the complete dataset DS1 (a), the two non‐outlier datasets, DS2 (b) and DS3 (c), and the two outlier datasets, DS4 (d) and DS5 (e). α‐Genetic diversity is estimated using the expected heterozygosity (*He*) and α‐species diversity by the wetland plant species richness and Pielou's evenness. β‐genetic dissimilarity is estimated using Cavalli‐Sforza genetic distance and β‐species dissimilarity is estimated using Bray–Curtis distance. S6 and S21 are the two outlier sites

Before calculating SGDCs, we generated scatterplots to check whether the genetic and species diversity data were linearly related. These scatterplots are provided in the Figure 2. α‐SGDCs were calculated as Pearson correlations between the plant α‐diversity estimates (i.e., plant richness and evenness) and *He* for each population and each of the five SNP datasets. We used *t* tests to test for the significance of the SGDCs. To test if, as expected, the SGDCs of the outlier loci datasets were significantly different than that of the nonoutlier loci datasets, we calculated the probability of obtaining such extreme SGDCs using an equal number of loci with DS1 and the putatively neutral datasets (DS2 and DS3). To this end, we calculated SGDC with 999 randomized subsets of DS1, DS2 and DS3 containing the same number of loci as DS4 and DS5. β‐SGDCs were investigated with Mantel tests using the pairwise genetic distance matrices and Bray–Curtis dissimilarity matrix of the plant species assemblage based on 9,999 permutations.

## 3 RESULTS

### 3.1 Genotyping

Illumina sequencing produced around 2.5 million reads per individual. Following basic filtering steps conducted in the UNEAK pipeline, we retained 38,036 SNPs. With the additional, more stringent filtering protocol, we obtained 1,709 high‐quality SNPs for our overall dataset (Supporting Information Table S2). This filtering process reduced our number of individuals from 190 to 158 and the number of wetlands from 20 to 17. In our final dataset, the mean depth per individual was 29.9 reads and the mean depth per site across all individuals was 30.0 reads. No loci were found to depart from Hardy–Weinberg equilibrium at an α = 0.05 significance level in more than 50% of the sampling locations. At the population level, no more than 2.5% of the loci, on average, were found to significantly deviate from Hardy–Weinberg equilibrium.

### 3.2 Outlier identification and datasets

In the scree plot of the PCA conducted in pcadapt, the elbow occurred at the 10th component. With a false discovery rate of 10%, 173 SNPs were identified as outliers.

After selecting the predictor variables according to their correlations, none of those included in the RDA analysis showed a VIF greater than 5. The RDA model explained 20.9% of the genetic variance. Based on the elbow of the eigenvalues scree plot, we retained the first four components for outlier detection. All components were highly significant, with axes 1 through 4 explaining 35%, 22%, 16%, and 9% of the explained variance, respectively, all together accounting for 82% of the variance explained by the RDA model. In total, 95 loci had outlier *z*‐scores on at least one of the four RDA components. Outliers were most strongly correlated with topographic slope on axis 1, with moisture‐related variables (TCI and MAP) on axis 2, moisture and wind speed (TCI and MAWS) on axis 3, and with temperature (MAT) and slope on axis 4.

RDA and pcadapt together identified a total of 229 outlier loci, of which 39 were identified in both methods (Table 2). They formed the two outlier datasets, DS4 and DS5, respectively. The two nonoutlier datasets, DS2 and DS3, were constructed by removing DS4 and DS5 datasets from DS1, respectively. After removal of outlier loci, DS2 and DS3 contained 1,480 and 1,670 loci, respectively.

### 3.3 Species and genetic diversity estimates

Species richness ranged between 10 and 21 across the sites, and Pielou's evenness between 0.39 and 0.82. The average expected heterozygosity for the full dataset (DS1) across all sites was 0.130. It reached 0.135 and 0.132 for DS2 and DS3, and 0.098 and 0.050 for DS4 and DS5 (Table 1). The α‐genetic diversity estimates (*He*) of DS1, DS2 and DS3 correlated almost perfectly (*r *>* *0.99, *p *<* *0.001 in all cases). While the *He* estimates of DS4 correlated with those of DS1, DS2, and DS3 (range: 0.63–0.70, *p *<* *0.02 in all cases), no such relationships were observed between DS5 and the putatively neutral datasets (range: −0.23 to −0.16, *p *=* *1.00 in all cases). The genetic diversity estimates calculated from the nonoutlier datasets (DS1, DS2, and DS3) and those obtained by Bertin et al. (2017) with AFLP markers were strongly correlated (*r* range: 0.68–0.70, *p *=* *0.02 in all cases). Overall, the genetic diversity estimates tended to be higher with the SNPs than with the AFLPs. Two sites, however (sites 6 and 21), departed strongly from the main relationship, showing unexpectedly low SNP genetic diversity compared to their respective AFLP estimates (Supporting Information Figure S3). By removing these two sites, the correlation between the SNP and AFLP genetic diversity increased substantially, from 0.70 to 0.90 with DS1. Both of these sites were sequenced jointly on lane 1. They presented a proportion of missing data of 5.5% and 1.8%, respectively. The percentage of missing data in site 21 appears lower than the average found for this lane in the postfiltered data (3.8%). Although the proportion found in site 6 is slightly higher, it is still lower than other sites that were also sequenced in lane 1, such as site 19 (with 8.4% missing data), and also similar to the average found for lane 2 (6%). Together, these results indicate no atypical behavior of the SNP data for these two sites that could be due to technical artifacts.

The genetic distances ($D_{\mathrm{CH}}$) calculated with DS1, DS2, and DS3 ranged between 0.03 and 0.15. In all three cases, site 19 stood out from the rest of the sites for exhibiting many high genetic distance values (mean $D_{\mathrm{CH}}$ values ranging between 0.11 and 0.12). While the $D_{\mathrm{CH}}$ varied between 0.02 and 0.19 for DS4, they reached much lower and higher extreme values for DS5, which ranged between 0.002 and 0.31. Yet, in both cases, site 21 was found to display noticeably high genetic distances compared to the rest of the sites (mean $D_{\mathrm{CH}}$ values: 0.14 and 0.24 for DS4 and DS5, respectively). Plant composition distances measured as Bray–Curtis distances ranged between 0.27 and 0.87. No site showed noticeable higher differentiation levels than the others, but all of them were highly differentiated from some other sites in terms of plant composition, with all sites showing a BC value of at least 0.67.

### 3.4 α‐Species–genetic diversity correlations with outlier and nonoutlier SNP loci

The α‐SGDCs estimated from species richness were positive for DS1 and the two nonoutlier datasets (DS2 and DS3). However, the correlations were only moderate in amplitude, ranging from 0.33 to 0.37, and marginally nonsignificant (*p *=* *0.10, 0.07, and 0.09 for DS1, DS2, and DS3, respectively, Table 2). These correlations were much lower than the SGDCs reported by Bertin et al. (2017). When excluding sites 6 and 21, however, the SGDC values increased substantially (more than 60%), with correlations ranging from 0.57 to 0.60 for datasets DS1–DS3, and became highly significant (Table 2). No significant positive correlations were observed for the outlier datasets (Table 2); rather, the correlations were negative and not statistically significant. The results of the randomizations show that it would be extremely rare to obtain α‐SGDCs as low as those observed with DS4 and DS5 just by chance with the putatively non‐neutral datasets (*p *≤* *0.001 in all cases).

None of the α‐SGDCs estimated with species evenness were significant (Table 2). In spite of that, the randomizations demonstrated that the estimates derived from DS4 differed significantly from those obtained with DS1, DS2, and DS3 (*p *≤* *0.01 in all cases). The putatively non‐neutral datasets displayed lower α‐SGDCs than the putatively neutral datasets, reaching −0.24 for DS4 and ranging between 0.01 and −0.07 for DS1, DS2, and DS3.

### 3.5 β‐Species–genetic diversity correlations with outlier and nonoutlier SNP loci

The β‐SGDCs were all positive. For the full and putatively neutral datasets (DS1‐3), they ranged between 0.11 and 0.16 and were not significant or marginally nonsignificant (*p* > 0.08, Table 2). For the putatively non‐neutral datasets (DS4–5), correlations varied from 0.21 to 0.27 and were significant (*p* < 0.05, Table 2). The results of the randomizations show that the probability of getting such high β‐SGDCs by chance would be rare, particularly with DS2 and DS3 (*p* < 0.03 in all cases, Supporting Information Table S3).

## 4 DISCUSSION

### 4.1 Partitioning genetic and species diversity in SGDCs studies

Recent studies have stressed the usefulness of partitioning neutral and adaptive genetic diversity (Bertin et al., 2017; Watanabe & Monaghan, 2017) and of simultaneously analyzing various species diversity components (Lamy et al., 2017) to investigate species–genetic diversity relationships. Our results show that combining these two approaches can help to unravel the origin of covariation between these two levels of diversity. As expected, we found contrasting SGDCs between the putatively neutral and non‐neutral datasets. Yet, the detected patterns diverged greatly depending on the species diversity component. Indeed, α‐SGDCs were detected with species richness but not with species evenness. And, while the α‐SGDCs based on species richness were only significant and stronger with the nonoutlier datasets compared to the outlier loci datasets, an opposite trend was observed for the β‐SGDCs.

While the SGDCs of the nonoutlier loci datasets were much weaker that those previously reported with AFLP data, they provide relevant information. For the α‐diversities, significant and positive SGDCs were only found with the nonoutlier loci datasets and with species richness, confirming that neutral processes were primarily driving the correlations and that the involved processes differentially influenced species richness and evenness. In their study, Bertin et al. (2017) examined the effects of wetland size, stability and connectivity on local diversities. They found that connectivity influenced plant species richness and *C. gayana* genetic diversity. We reproduced this analysis with species evenness but failed to detect such effects (results not shown). This finding is consistent with the expectation that migration more strongly influences species richness than species evenness (Wilsey & Stirling, 2007) and supports a role for migration rates in the detected α‐SGDCs. So far, few empirical studies have combined species evenness and richness to investigate species–genetic diversity relationships. Of the 161 α‐SGDCs gathered by Lamy et al. (2017), only 18 were calculated with some kind of evenness index, and just eight studies used species richness and evenness in calculating SGDCs for the same dataset and genetic measure, reporting in most cases no significant SGDCs for both indices. Our results suggest that it can be profitable to simultaneously analyze the two diversity components as they may reveal different patterns and thus help understand mechanisms behind species–genetic diversity relationships.

The β‐SGDCs with the putatively neutral datasets were lower than the α‐SGDCs obtained with species richness, which is consistent with the trends described by Lamy et al. (2017). However, while Lamy et al. (2017) found that β‐SGDCs are more often significant than α‐SGDCs, none of our β‐SGDCs with the putatively neutral datasets were significant (at α = 0.05). Significant β‐SGDCs were detected with the putatively non‐neutral loci. The contrasting patterns in β‐SGDCs between the putatively neutral and non‐neutral datasets thus indicate that selective processes influencing *C. gayana* genetic diversity are involved in the detected correlation, which does not exclude the possibility that neutral processes are also contributing to it. Separating β‐genetic diversity into putatively neutral and non‐neutral components can help elucidate which processes are influencing the genetic level of the β‐SGDCs but does not provide information about the ecological processes involved in community dissimilarity. We see two possible alternatives. First, the concordance in genetic and species composition dissimilarities resulted from common responses to environmental variation. Most of the environmental predictors that contributed to the detection of outliers in the genotype‐by‐environment analysis (i.e., moisture, precipitation, wind speed, temperature, and slope) can be linked to drought, which may be a key factor structuring the composition of vegetal communities in high Andean wetlands (Dangles et al., 2017). Alternatively, β‐SGDCs can arise from different processes affecting composition dissimilarities among sites at the genetic and community levels, as long as the spatial scales at which they are acting are similar. This would be the case, for instance, if distance decay patterns generated by environmental gradients on genetic dissimilarity match distance decay patterns generated by dispersal limitation at the community levels. A better understanding of the origin of β‐SGDCs could be gained by decomposing the correlation into underpinning factors as proposed by Lamy et al. (2017), but such an approach requires a large number of sites (Lamy et al., 2017).

### 4.2 The usefulness and the limits of SNP markers for partitioning neutral and adaptive genetic diversity in SGDC studies

Our work extends and refines previous studies that suggested investigating neutral and adaptive genetic diversity simultaneously to reveal neutral signatures in species–genetic diversity relationships (Bertin et al., 2017; Watanabe & Monaghan, 2017) and shows that this framework can be successfully applied in single‐species studies when large genomic datasets are available. The efficiency of such approach, however, depends on how much the adaptive and neutral genetic diversity deviate from each other, and may thus be better suited for studies focusing on fragmented ecosystems occurring over environmental gradient, as was the case here. The presence of false positives in the outlier datasets may also be another limiting factor, but this problem is likely to be resolved in the near future since it will be possible to have more genomic resources for nonmodel species (e.g., fully sequenced and annotated genomes) that will allow the validation of outlier loci as candidate genes for adaptation (Manel et al., 2016).

Markers traditionally used in SGDC studies such as simple sequence repeats (SSR) and AFLPs are of limited use in teasing apart the neutral and non‐neutral components of genetic diversity because these markers sample only a small proportion of the entire genome and usually produce a relatively limited number of markers, hampering the identification of numerous outlier loci. However, the combined information of AFLP and SNP data provided unexpected results. Overall, we found slightly higher genetic diversity estimates with the 1,709 SNPs than with the 85 AFLP data in all but two sites, which departed from this general trend and presented conspicuously low SNP genetic diversity estimates compared to their AFLP counterparts (sites 6 and 21, Supporting Information Figure S3). We tested whether the low SNP estimates in these two sites could be due to sampling effects by re‐calculating the AFLP genetic diversity using only the individuals considered for the SNP estimation. No matching trend was detected. Given that these two sites belong to the same cpDNA lineage and AFLP cluster as the other sites in this study (as described in Troncoso et al., 2017), this discrepancy cannot be explained by a higher level of allelic dropout in these sites potentially caused by strong differences in their genetic composition compared to the other sites. Because technical issues are also very unlikely, the incongruent results between SNPs and AFLPs for those two sites suggest that evolutionary processes reducing genetic diversity may have affected these two marker types differently. SNPs have low mutation rates (10 × 10^−8^ to 10 × 10^−9^; Nachman & Crowell, 2000), much lower than microsatellites (0.001 to 0.005; Pinto et al., 2013), whereas AFLP mutation rates can exceed those of microsatellites (Kuchma, Vornam, & Finkeldey, 2011). As a consequence, AFLPs respond more strongly to recent demographic events than SNPs, whose polymorphisms may actually reflect the effects of recent to intermediate evolutionary events (Waits & Storfer, 2016). The possibility of different responses by AFLPs and SNPs is supported by the recent comparison of genetic diversities in *Arabidopsis* between SNP and microsatellites (Fischer et al., 2017). The relatively low SNP genetic diversity observed in sites 6 and 21 could therefore indicate a reduction in genetic diversity in these two *C. gayana* populations due to a past demographic event, whose effects could no longer be detected in AFLPs.

The SNP genetic diversity of these two sites reduced the species–genetic diversity relationship with species richness, and once removed, the SGDCs with the full and nonoutlier datasets were much higher (and closer to the value reported in Bertin et al., 2017). This suggests that SNP markers responded uniquely to some evolutionary processes in sites 6 and 21. Genes versus species may differ in their rates of response to evolutionary pressures, and the responses of species and genetic diversity may not always be equally strong. Our results suggest that SNPs could strongly respond and show longer‐lasting response to diversity‐reducing processes such as drift than AFLP markers.

## AUTHOR CONTRIBUTIONS

NG and AB acquired and processed data. BF filtered the SNP dataset. BF, JH, AM, and VP conducted genetic structure and outlier analyses. JH, JLBP, and AB conducted regression analysis. VP, BF, JH, AM, NG, and AB drafted the manuscript. JLBP, AB, and VP produced figures. AB, NG, and SM guided the conceptual development of the manuscript. All authors contributed to revisions and approved the final version of the article.

## DATA ARCHIVING

Raw files of the SNP data will be made available from the Dryad Digital Repository upon acceptance of the manuscript.
