# Supplementary material for: Partitioning genetic and species diversity refines our understanding of species–genetic diversity relationships
Source: Ecol Evol. 2018 Dec 11;8(24):12351–64. doi: 10.1002/ece3.4530 (PMC6308885; doi:10.1002/ece3.4530)
Supplement: Supplementary file 1 [file ECE3-8-12351-s001.docx]

**Supplementary Material**

**SNP markers refine our understanding of species-genetic diversity relationships**

Vera Pfeiffer, Brett Ford, Johann Housset, Audrey McCombs, José Luis Blanco-Pastor, Nicolas Gouin, Stéphanie Manel and Angéline Bertin

**Table S1**: Summary of the key commands used in the UNEAK pipeline at the Genomic Diversity Facility of the Cornell University.

| Plugin | Option | Value | Description |
| --- | --- | --- | --- |
| FastqToTagCountPlugin | c | 1 | Minimum number of times a tag must be present to be output. Default: 1 |
| FastqToTagCountPlugin | s | 300000000 | Max good reads per lane. (Optional.  Default is 300000000). |
| MergeMultipleTagCountPlugin | c | 3 | Minimum number of times a tag must be present to be output. Default: 1 |
| TagCountToFastqPlugin | c | 1 | Minimum count of reads for a tag to  be output (default: 1) |
| UTagCountToTagPairPlugin | e | 0.03 | Error tolerance rate in the network filter. (Default: 0.03) |
| UExportTagPairPlugin | d | 1000 | (or --distance) distance to pad tag  pairs by (default: 1000) |
| FastqToTBTPlugin | y | -y | output to tagsByTaxaByte (tag counts per taxon from 0 to 127) instead of tagsByTaxaBit (0 or 1) |
| FastqToTBTPlugin | c | 1 | Minimum taxa count within a qseq  file for a tag to be output. Default: 1 |
| MergeTagsByTaxaFilesPlugin | s | 300000000 | Maximum number of tags the TBT can hold while merging (default:  200000000) |
| TagsToSNPByAlignmentPlugin | y | -y | Use byte-formatted TBT file (*.tbt.byte) |
| TagsToSNPByAlignmentPlugin | errRate | 0.01 | Average sequencing error rate per base (used to decide between heterozygous and homozygous calls). Default: 0.01 |
| TagsToSNPByAlignmentPlugin | mnLCov | 0.1 | Minimum locus coverage i.e. the  proportion of taxa with at least one  tag at the locus. Default: 0.1 |
| TagsToSNPByAlignmentPlugin | mxSites | 2000000 | The maximum number of SNPs per  chromosome for hapmap files (default = 2000000) |
| TagsToSNPByAlignmentPlugin | mnMAC | 999 | Minimum minor allele count. Defaults to 10. SNPs that pass either the specificed minimum minor allele count (mnMAC) or frequency (mnMAF) will be output. |
| TagsToSNPByAlignmentPlugin | mnMAF | 0.01 | Minimum minor allele frequency.  Defaults to 0.01. SNPs that pass either the specifice dminimum minor allele frequency (mnMAF) or count (mnMAC) will be output. |

| Plugin | Option | Value | Description |
| --- | --- | --- | --- |
| MergeDuplicateSNPsPlugin | misMat | 0.05 | Threshold mismatch rate above which the duplicate SNPs won’t be merged. Default: 0.05. But if residual heterozygosity is expected (not inbred lines) then it should be set to 0.1-0.2 |
| MergeDuplicateSNPsPlugin | callHets | -callHets | When two genotypes at a replicate  SNP disagree for a taxon call it a  heterozygote. Defaults to false (=set  to missing) |
| FastqToTBTPlugin | y | -y | Output to tagsByTaxaByte (tag counts  per taxon from 0 to 127) instead of  tagsByTaxaBit (0 or 1) |
| FastqToTBTPlugin | c | 1 | Minimum taxa count within a qseq  file for a tag to be output. Default: 1 |
| MergeTagsByTaxaFilesPlugin | s | 300000000 | Maximum number of tags the TBT can  hold while merging (default:  200000000) |
| tbt2vcfPlugin | ak | 3 | Maximum number of alleles that are  kept for each marker across the  population default: 3 |
| tbt2vcfPlugin | mnLCov | 0.0 | Minimum locus coverage (proportion  of Taxa with a genotype) (default: 0.0) |
| tbt2vcfPlugin | mnMAF | 0.0 | Minimum minor allele frequency  (default: 0.0) |
| MergeDuplicateSNP_vcf_Plugin | ak | 3 | Maximum number of alleles that are  kept for each marker across the  population default: 3 |
| GBSHapMapFiltersPlugin | mnSCov | 0.8 | Minimum site coverage (default: no  filter) |
| GBSHapMapFiltersPlugin | mxMAF | 1 | Maximum minor allele frequency  (default: 1.0 = no filter) |
| GBSHapMapFiltersPlugin | mnTCov | 0.1 | Minimum taxa coverage (default: no  filter) |
| GBSHapMapFiltersPlugin | mnMAF | 0.01 | Minimum minor allele frequency. Default: 0.0 = no filter |

**Table S2:** The number of remaining variable sites after each filtering step in VCFtools (Danecek et al., 2011). The final number of loci used for the ‘full’ dataset (DS1) is indicated in bold.

| Filtering Step | VCFtools argument | Remaining Sites |
| --- | --- | --- |
| UNEAK GBS pipeline |  | 38036 |
| Minimum depth filter of 10 reads | --minDP 10 | 38036 |
| Minimum mean depth of 4 reads | --min-meanDP 4 | 9485 |
| Maximum mean depth of 50 reads | --max-meanDP 50 | 9082 |
| Maximum missing data at 40% | --max-missing 0.6 | 3007 |
| Minimum allele frequency at 0.04 | --maf 0.04 | 1926 |
| Bialleleic loci | --min-alleles 2 --max-alleles 2 | 1926 |
| Exclude loci with H_O_ >0.5 | --exclude-positions | 1709 |
| Call rate > 40% | --remove | **1709 sites for 158 individuals** |

**Table S3**: Probabilities of the SGDC values observed with the putatively non-neutral datasets (DS4 and DS5) calculated with 999 randomized subsets of DS1, DS2 and DS3 containing an equal number of loci than DS4 and DS5.

|  |  | Probability of the observed SGDC for | | | |
| --- | --- | --- | --- | --- | --- |
|  |  | DS4  Number of loci = 229 | | DS5  Number of loci = 39 | |
|  |  | 17 sites | 15 sites | 17 sites | 15 sites |
| α-SGDC with species richness | DS1 | <0.001 | <0.001 | 0.001 | 0.001 |
|  | DS2 | <0.001 | <0.001 | <0.001 | <0.001 |
|  | DS3 | <0.001 | <0.001 | <0.001 | <0.001 |
| α-SGDC with species evenness | DS1 | 0.008 | 0.002 | 0.23 | 0.08 |
|  | DS2 | <0.001 | <0.001 | 0.17 | 0.06 |
|  | DS3 | 0.002 | 0.001 | 0.21 | 0.08 |
| β-SGDC | DS1 | <0.001 | <0.001 | 0.08 | 0.01 |
|  | DS2 | <0.001 | <0.001 | 0.03 | 0.003 |
|  | DS3 | <0.001 | <0.001 | 0.05 | 0.005 |

**Figure S1**. Effects of missing data on genetic diversity estimates. Expected heterozygosity was calculated for each of the 17 populations (represented with various colors) using only loci that met a minimum threshold of genotyped individuals varying from one to eight.


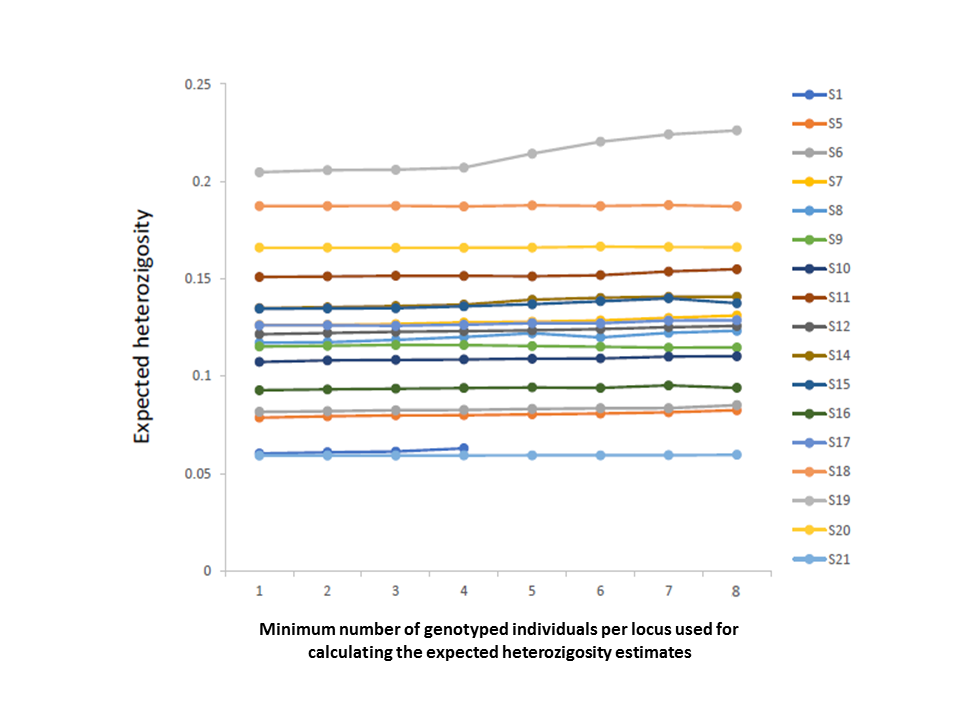


**Figure S2.** Scatterplots of the expected heterozygosity of the *Carex gayana* populations computed with the different SNP datasets (DS1: full SNP dataset; DS2 and DS3: the putatively neutral datasets and DS4 and DS5: the putatively adaptive datasets.

**
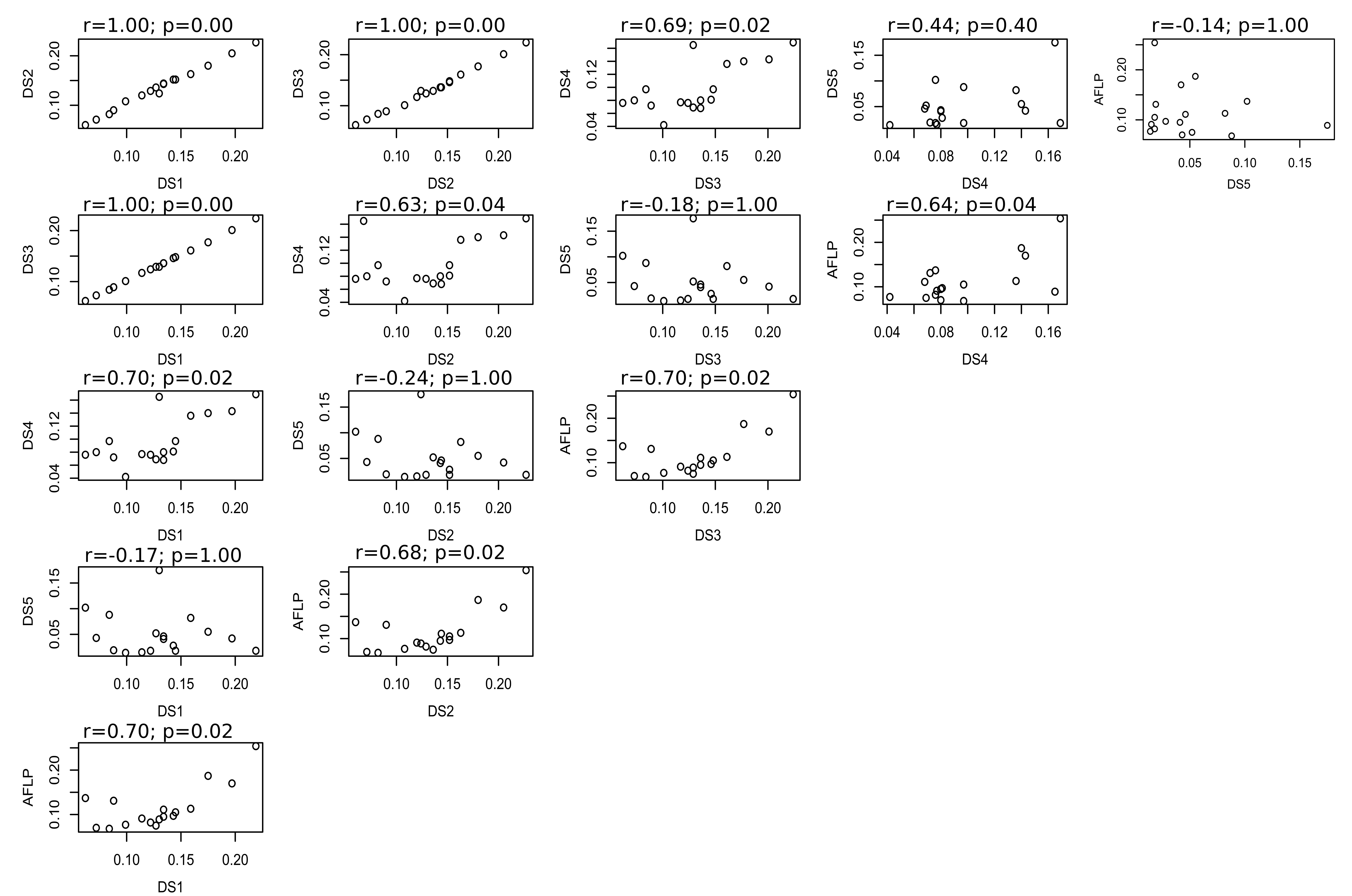
**

**Figure S3.** Scatterplot of the expected heterozygosity of the *Carex gayana* populations computed with SNPs vs AFLP markers. Filled circles show AFLP genetic diversity computed on the exact same individuals used for the SNP dataset, triangles show AFLP genetic diversity computed with all the original individuals from Bertin et al. (2017). Color gradient represents species diversity observed at sampling locations.


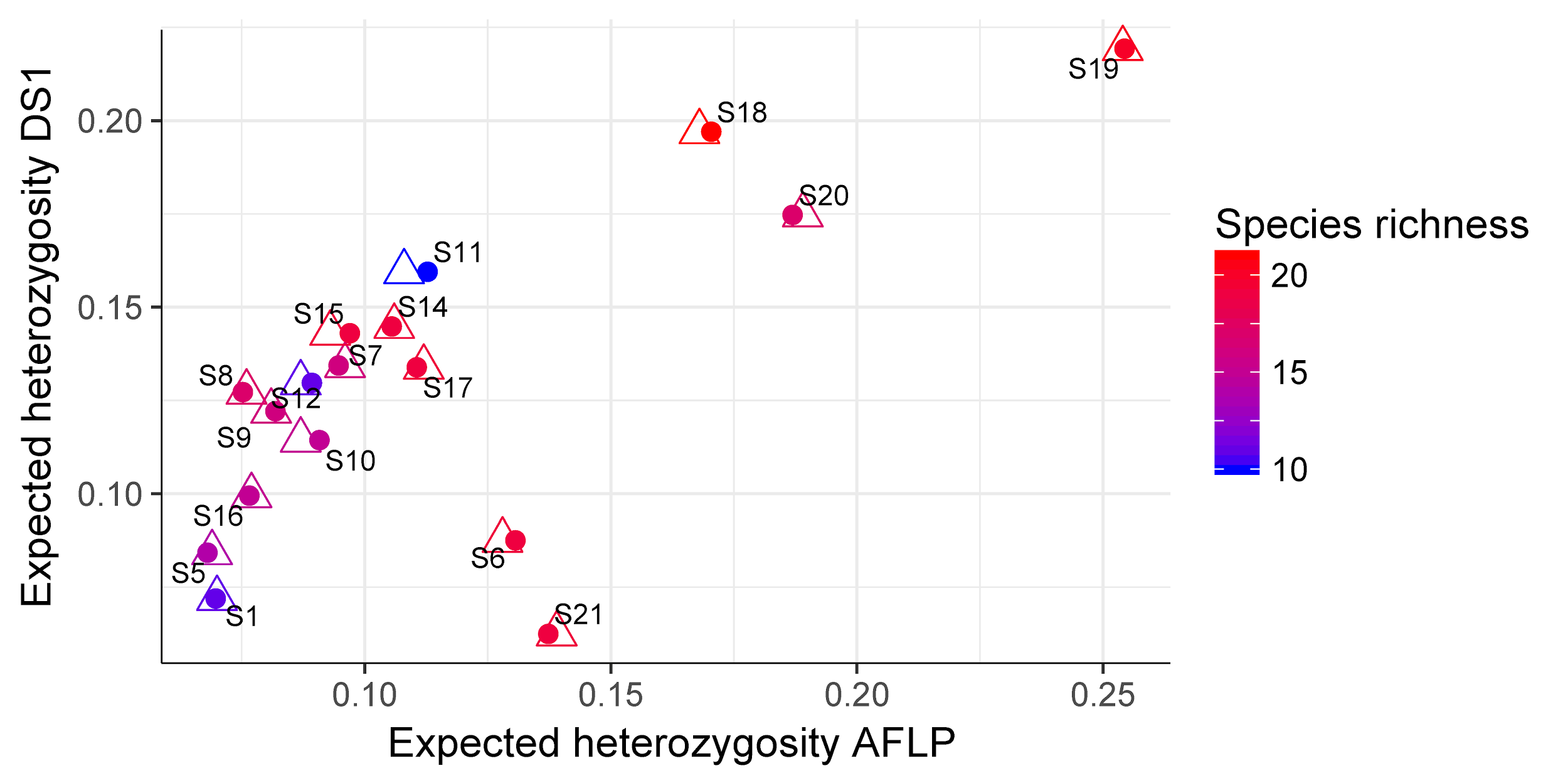


**SUPPLEMENTARY MATERIAL REFERENCES**

BERTIN, A., GOUIN, N., BAUMEL, A., GIANOLI, E., SERRATOSA, J., OSORIO, R. & MANEL, S. 2017. Genetic variation of loci potentially under selection confounds species–genetic diversity correlations in a fragmented habitat. *Molecular Ecology,* 26**,** 431-443.

DANECEK, P., AUTON, A., ABECASIS, G., ALBERS, C. A., BANKS, E., DEPRISTO, M. A., HANDSAKER, R. E., LUNTER, G., MARTH, G. T., SHERRY, S. T., MCVEAN, G., DURBIN, R. & GROUP, G. P. A. 2011. The variant call format and VCFtools. *Bioinformatics,* 27**,** 2156-2158.
